# Supplementary material for: Behavioral dysfunction but not wasting in cancer cachexia is driven by altered tryptophan metabolism
Source: bioRxiv. 2026 Oct 9:2026.10.08.757616. Preprint. [Version 1] doi: 10.64898/2026.10.08.757616 (PMC13692228; doi:10.64898/2026.10.08.757616)
Supplement: Supplementary file 1 [file NIHPP2026.10.08.757616v1-supplement-2.pdf]

bioRxiv preprint doi: https://doi.org/10.64898/2026.10.08.757616; this version posted October 9, 2026. The copyright holder for this preprint (which was not certified by peer review) is the author/funder, who has granted bioRxiv a license to display the preprint in perpetuity. It is made available under a CC-BY 4.0 International license.

Figure S1

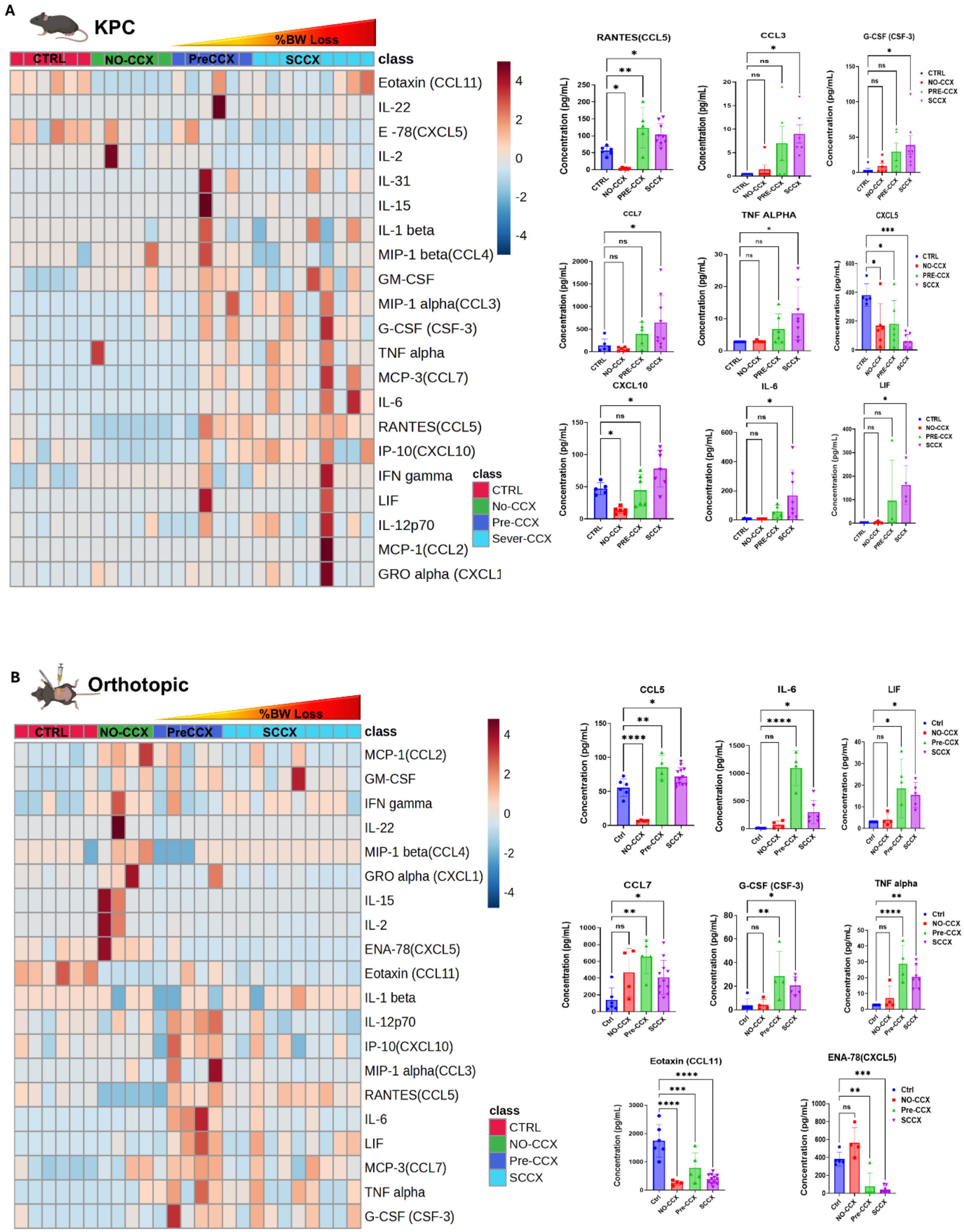

bioRxiv preprint doi: https://doi.org/10.64898/2026.10.08.757616; this version posted October 9, 2026. The copyright holder for this preprint (which was not certified by peer review) is the author/funder, who has granted bioRxiv a license to display the preprint in perpetuity. It is made available under a CC-BY 4.0 International license.

Figure S2

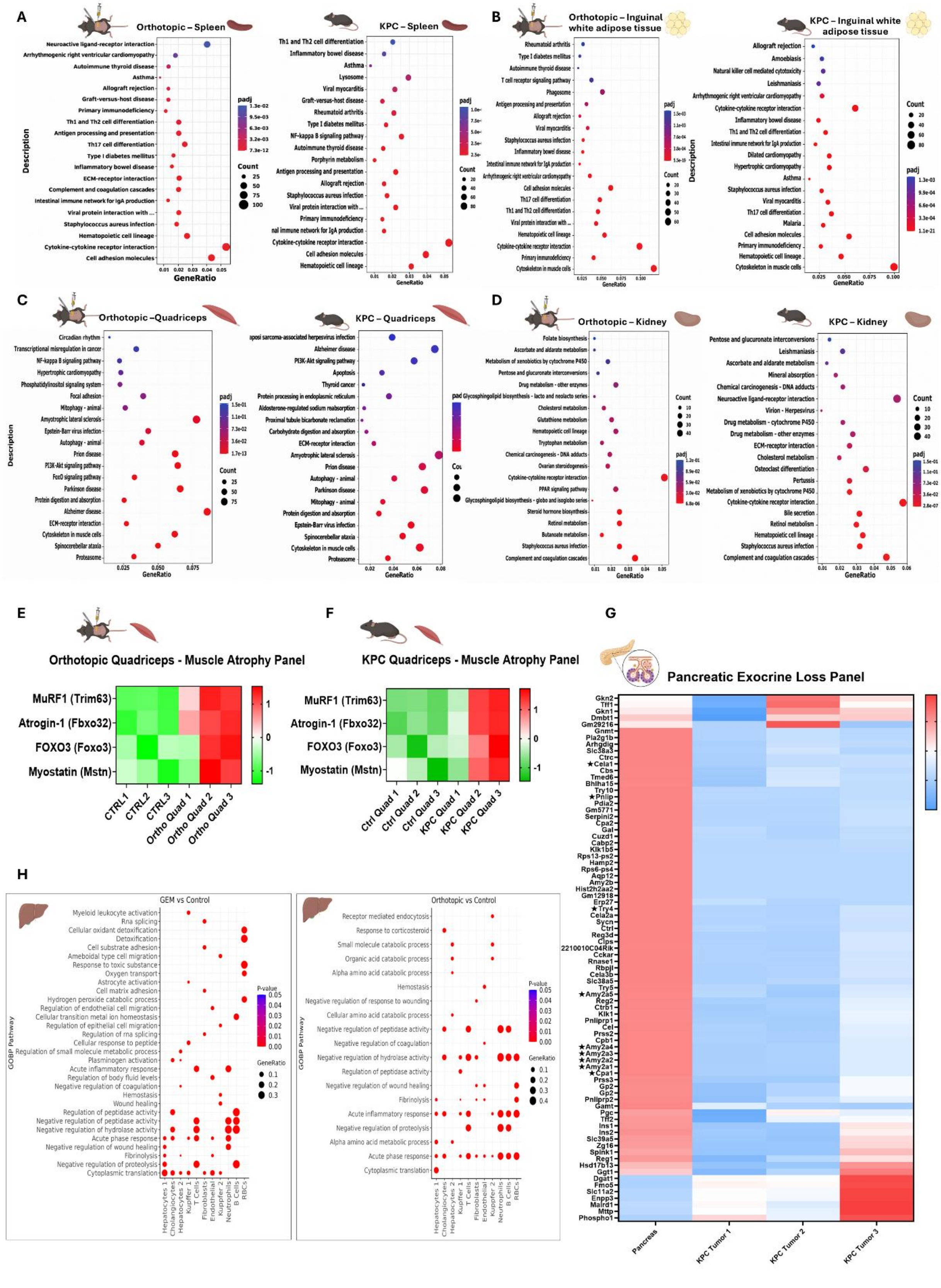

bioRxiv preprint doi: https://doi.org/10.64898/2026.10.08.757616; this version posted October 9, 2026. The copyright holder for this preprint (which was not certified by peer review) is the author/funder, who has granted bioRxiv a license to display the preprint in perpetuity. It is made available under a CC-BY 4.0 International license.

Figure S3

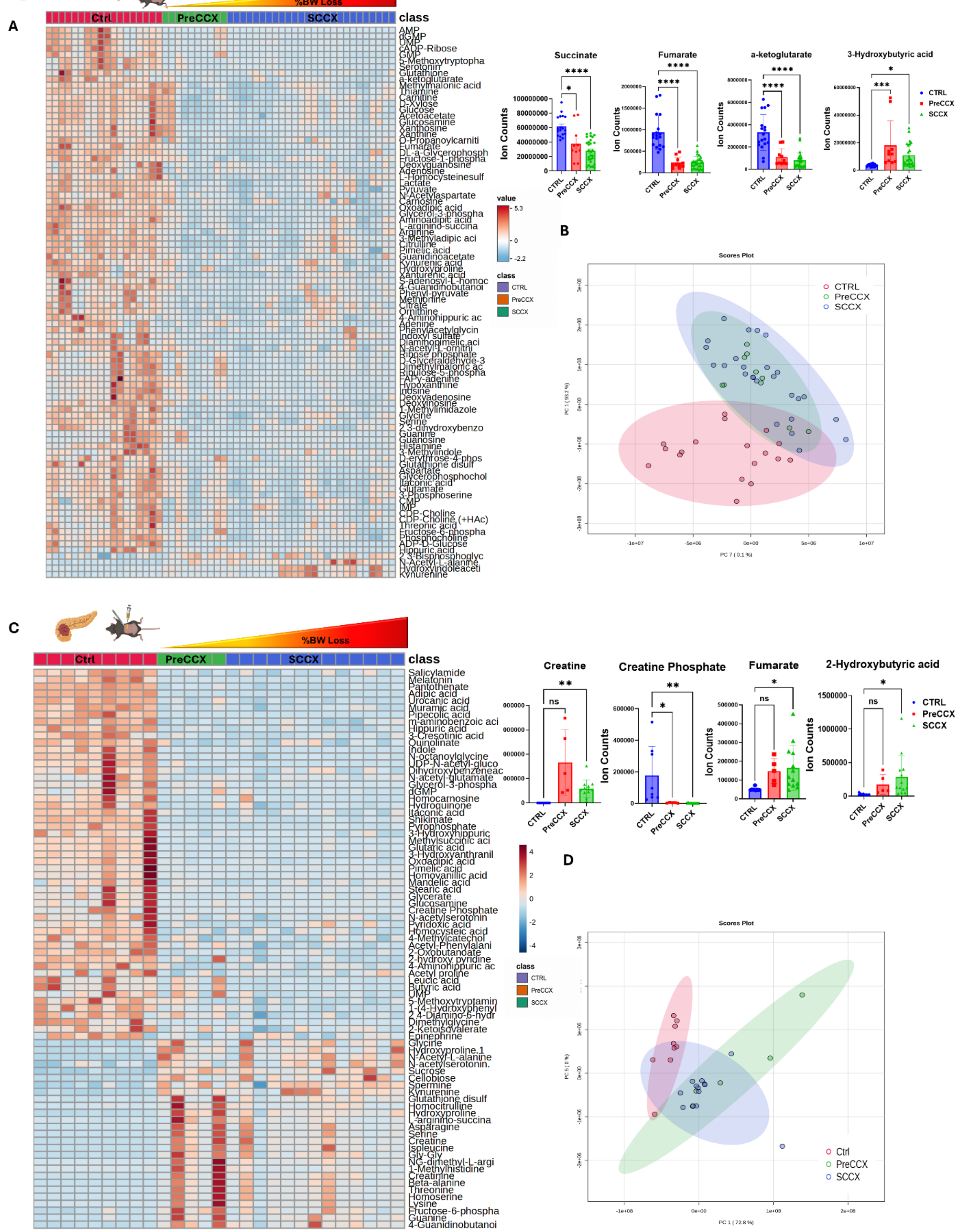

bioRxiv preprint doi: https://doi.org/10.64898/2026.10.08.757616; this version posted October 9, 2026. The copyright holder for this preprint (which was not certified by peer review) is the author/funder, who has granted bioRxiv a license to display the preprint in perpetuity. It is made available under a CC-BY 4.0 International license.

Figure S4

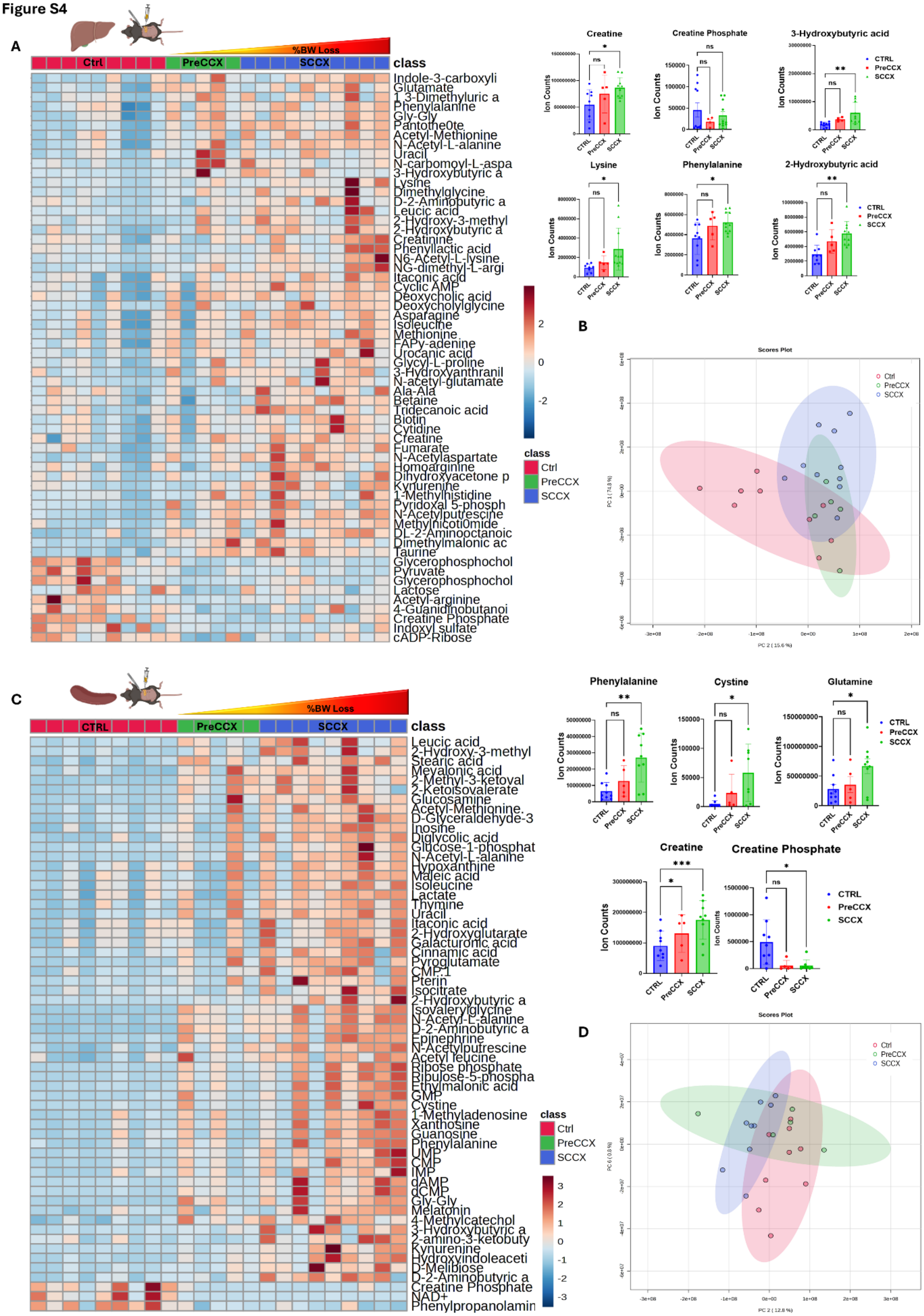

bioRxiv preprint doi: https://doi.org/10.64898/2026.10.08.757616; this version posted October 9, 2026. The copyright holder for this preprint (which was not certified by peer review) is the author/funder, who has granted bioRxiv a license to display the preprint in perpetuity. It is made available under a CC-BY 4.0 International license.

Figure S5

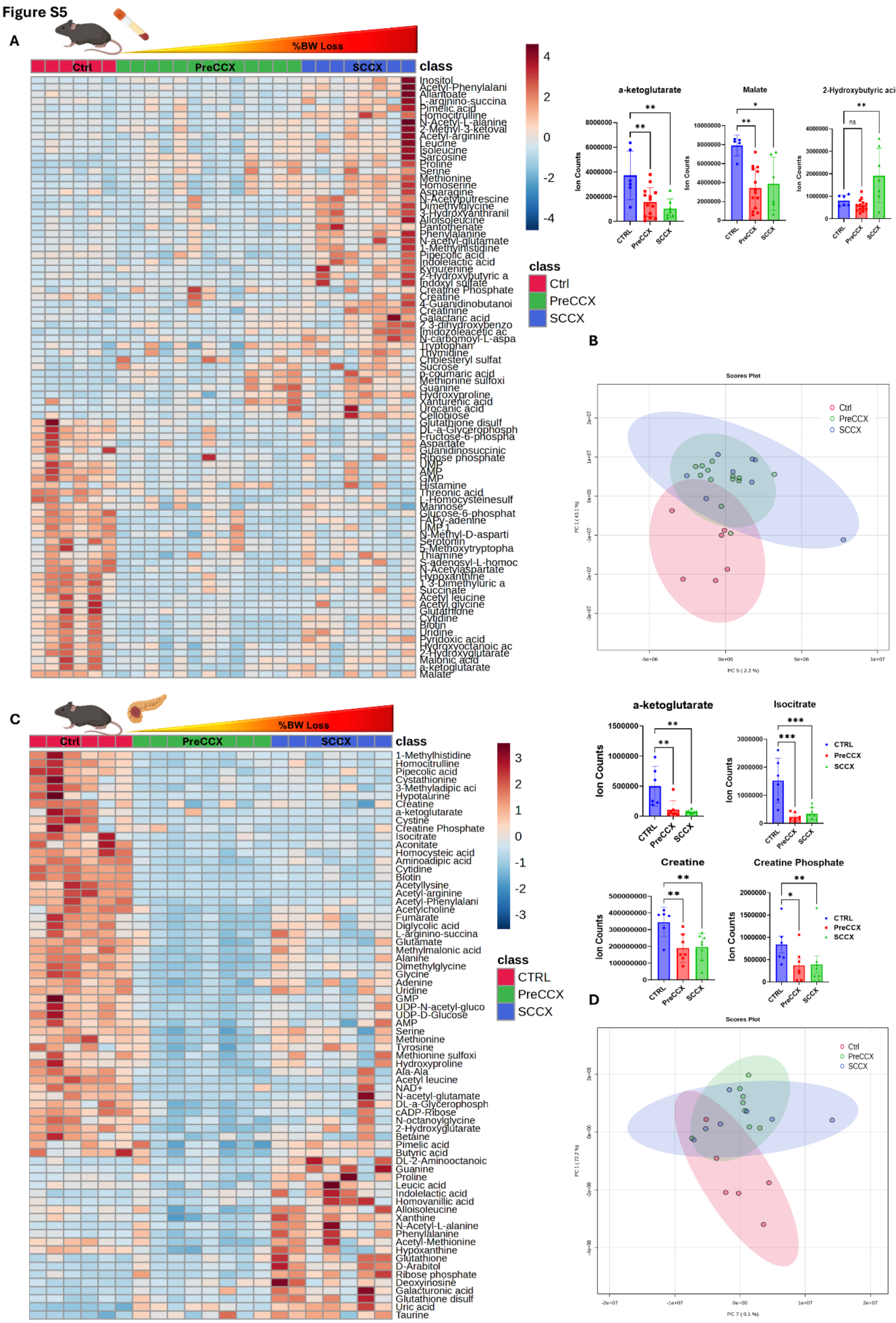

bioRxiv preprint doi: https://doi.org/10.64898/2026.10.08.757616; this version posted October 9, 2026. The copyright holder for this preprint (which was not certified by peer review) is the author/funder, who has granted bioRxiv a license to display the preprint in perpetuity. It is made available under a CC-BY 4.0 International license.

Figure S6

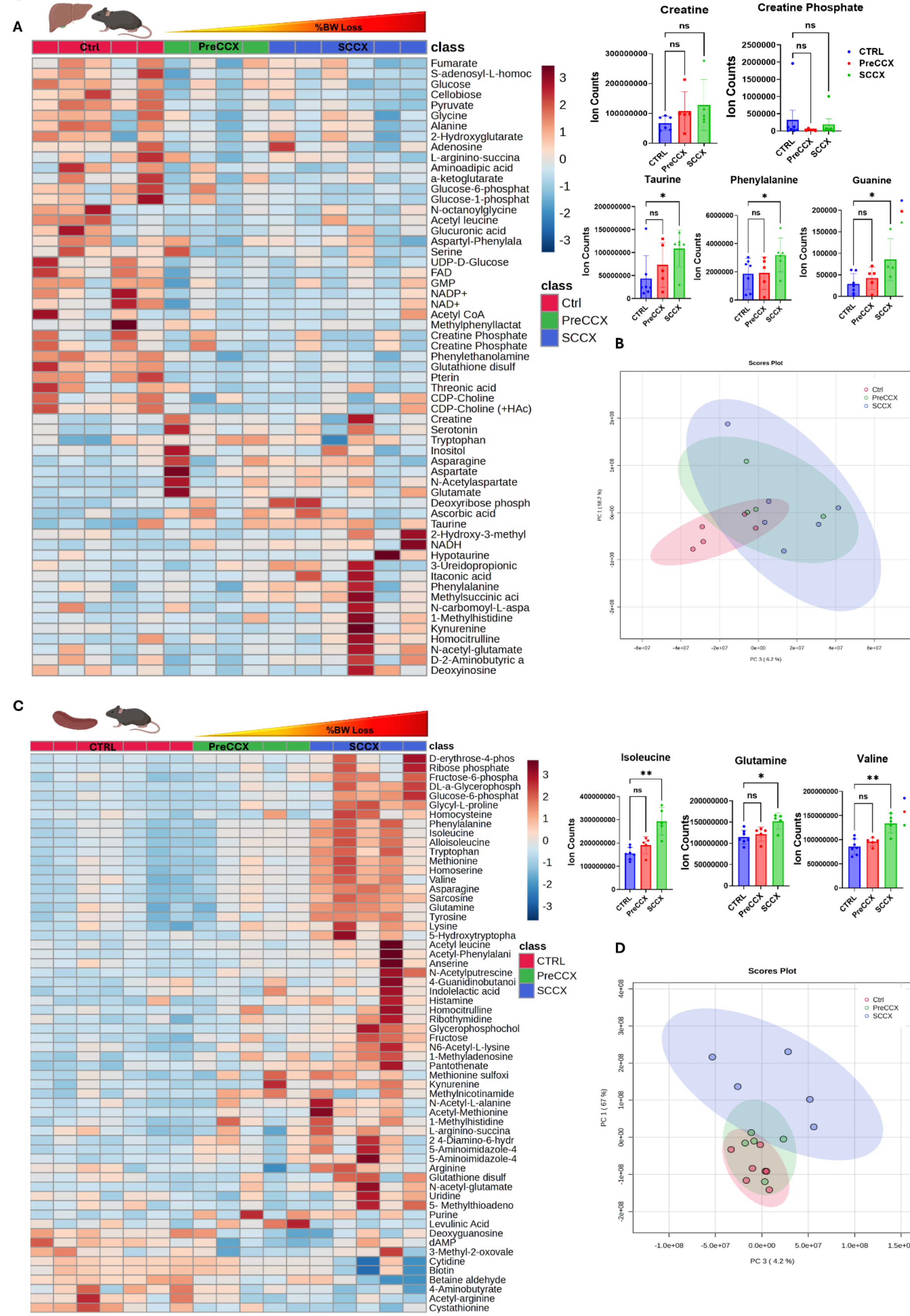

bioRxiv preprint doi: https://doi.org/10.64898/2026.10.08.757616; this version posted October 9, 2026. The copyright holder for this preprint (which was not certified by peer review) is the author/funder, who has granted bioRxiv a license to display the preprint in perpetuity. It is made available under a CC-BY 4.0 International license.

Figure S7

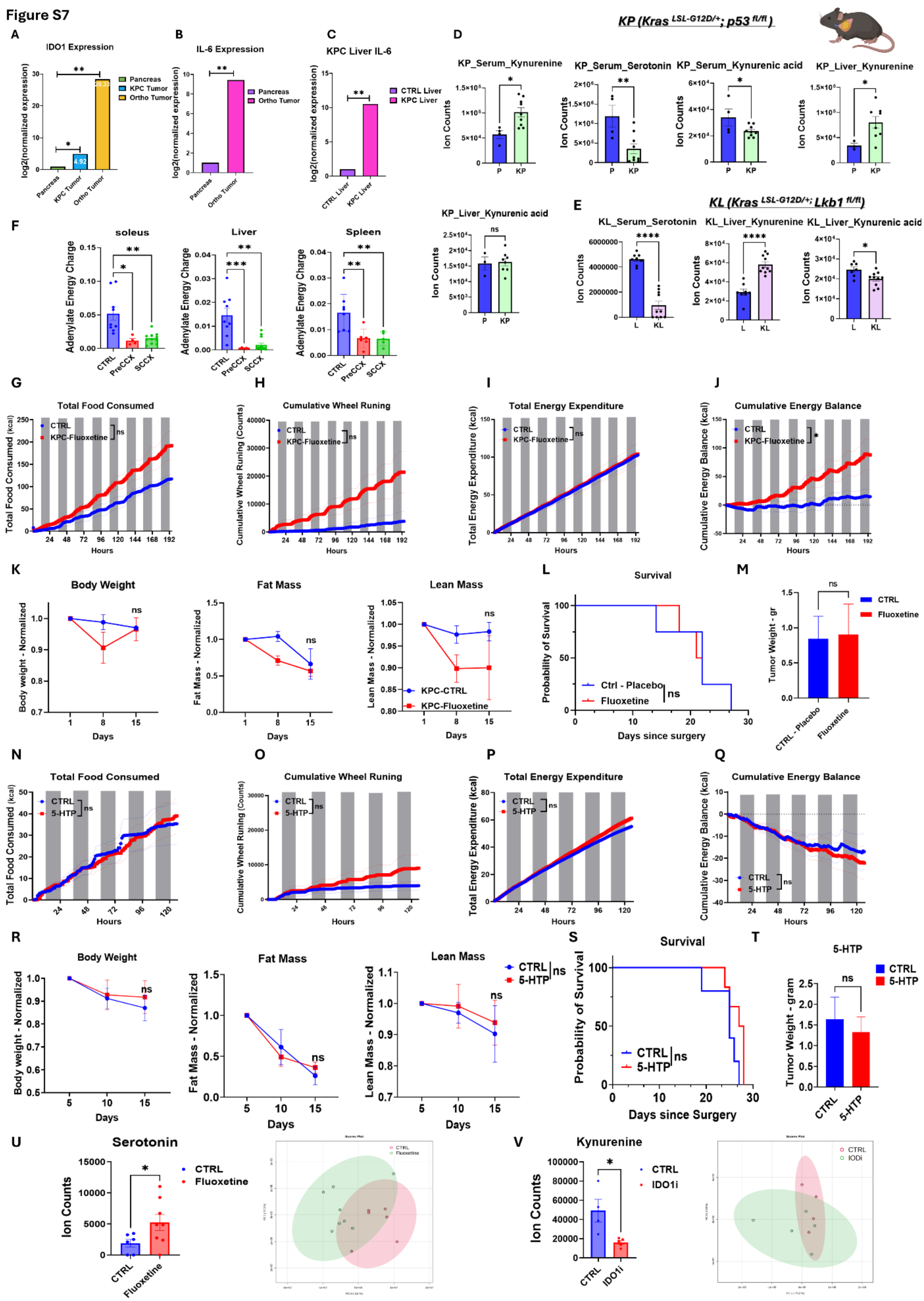

1182

bioRxiv preprint doi: https://doi.org/10.64898/2026.10.08.757616; this version posted October 9, 2026. The copyright holder for this preprint (which was not certified by peer review) is the author/funder, who has granted bioRxiv a license to display the preprint in perpetuity. It is made available under a CC-BY 4.0 International license.

Figure S1. Circulating inflammatory profiles across stages of PDAC cachexia, related to Figure 1.

(A) Circulating cytokine and chemokine profiles in control (CTRL; n = 6), non-cachectic (NO-CCX; n = 6), pre-cachectic (PreCCX; n = 6), and severe cachexia (SCCX; n = 9) GEMM KPC mice, shown as a heatmap with selected analytes.

(B) Corresponding profiles in orthotopic PDAC mice: CTRL (n = 6), NO-CCX (n = 4), PreCCX (n = 5), and SCCX (n = 13).

NO-CCX, non-cachectic; PreCCX, pre-cachectic; SCCX, severe cachexia; CCX, cachexia. Data are mean ± SEM. Individual analytes were analyzed by ordinary one-way ANOVA with Dunnett's multiple-comparisons test versus CTRL. Undetected analytes were excluded; therefore, n varies by analyte. *p < 0.05, **p < 0.01, ***p < 0.001, ****p < 0.0001; ns, p > 0.05.

Figure S2. Tissue transcriptional changes across PDAC cachexia models, related to Figure 2.

(A–D) Pathway enrichment of bulk RNA-seq profiles from orthotopic PDAC and GEMM KPC mice versus corresponding controls in spleen (A), inguinal white adipose tissue (B), quadriceps muscle (C), and kidney (D) (n = 3 mice per group). Dot size indicates gene count; color indicates adjusted p value.

(E and F) Expression of selected muscle atrophy-associated genes in quadriceps from orthotopic PDAC-bearing (E) and GEMM KPC (F) mice versus controls (n = 3 mice per group).

(G) Heatmap of pancreatic exocrine-associated gene expression across control and PDAC samples.

(H) Cell type-resolved Gene Ontology enrichment from liver single-nucleus RNA-seq in GEMM KPC and orthotopic PDAC-bearing mice versus corresponding controls (n = 3 mice per group).

bioRxiv preprint doi: https://doi.org/10.64898/2026.10.08.757616; this version posted October 9, 2026. The copyright holder for this preprint (which was not certified by peer review) is the author/funder, who has granted bioRxiv a license to display the preprint in perpetuity. It is made available under a CC-BY 4.0 International license.

Figure S3. Metabolomic profiles during orthotopic PDAC cachexia progression, related to Figure 3.

(A) Serum metabolite heatmap from control (CTRL; n = 18), pre-cachectic (PreCCX; n = 10), and severe cachexia (SCCX; n = 26) mice, with selected metabolites shown individually.

(B) Principal-component analysis (PCA) of serum metabolomic profiles.

(C) Pancreas/tumor metabolite heatmap from CTRL (n = 9), PreCCX (n = 5), and SCCX (n = 13) mice, with selected metabolites shown individually.

(D) PCA of pancreas/tumor metabolomic profiles.

PreCCX, pre-cachectic; SCCX, severe cachexia; CCX, cachexia. Data are mean ± SEM where applicable. Selected metabolites were analyzed by ordinary one-way ANOVA with Dunnett's multiple-comparisons test versus CTRL. Undetected metabolites were excluded; therefore, n varies by metabolite. Heatmaps include samples with complete metabolomic profiles; Metabolites not detected in a given sample were excluded from analysis; therefore, n varies by metabolite bar plots include all samples in which the indicated metabolite was measured. *$p < 0.05$, **$p < 0.01$, ***$p < 0.001$, ****$p < 0.0001$; ns, $p > 0.05$.

Figure S4. Liver and spleen metabolomic profiles during orthotopic PDAC cachexia progression, related to Figure 3.

(A) Liver metabolite heatmap from control (CTRL; n = 9), pre-cachectic (PreCCX; n = 5), and severe cachexia (SCCX; n = 9) mice, with selected metabolites shown individually.

(B) Principal-component analysis (PCA) of liver metabolomic profiles.

(C) Spleen metabolite heatmap from CTRL (n = 9), PreCCX (n = 5), and SCCX (n = 10) mice, with selected metabolites shown individually.

(D) PCA of spleen metabolomic profiles.

PreCCX, pre-cachectic; SCCX, severe cachexia; CCX, cachexia. Data are mean ± SEM where applicable. Selected metabolites were analyzed by ordinary one-way ANOVA with Dunnett's multiple-comparisons test versus CTRL. Undetected metabolites were excluded; therefore, n varies by metabolite. Heatmaps include samples with complete metabolomic profiles; Metabolites not detected in a given sample were excluded from analysis; therefore, n varies by metabolite bar plots include all samples in which the indicated metabolite was measured. *$p < 0.05$, **$p < 0.01$, ***$p < 0.001$, ****$p < 0.0001$; ns, $p > 0.05$.

bioRxiv preprint doi: https://doi.org/10.64898/2026.10.08.757616; this version posted October 9, 2026. The copyright holder for this preprint (which was not certified by peer review) is the author/funder, who has granted bioRxiv a license to display the preprint in perpetuity. It is made available under a CC-BY 4.0 International license.

Figure S5. Circulating and pancreatic/tumor metabolic remodeling during GEMM KPC cachexia progression, related to Figure 3.

(A) Serum metabolite heatmap from control (CTRL; n = 6), pre-cachectic (PreCCX; n = 13), and severe cachexia (SCCX; n = 8) GEMM KPC mice, with selected metabolites shown individually.

(B) Principal-component analysis (PCA) of serum metabolomic profiles.

(C) Pancreas/tumor metabolite heatmap from CTRL (n = 6), PreCCX (n = 8), and SCCX (n = 7) mice, with selected metabolites shown individually.

(D) PCA of pancreas/tumor metabolomic profiles.

PreCCX, pre-cachectic; SCCX, severe cachexia; CCX, cachexia. Data are mean ± SEM where applicable. Selected metabolites were analyzed by ordinary one-way ANOVA with Dunnett's multiple-comparisons test versus CTRL. Undetected metabolites were excluded; therefore, n varies by metabolite. Heatmaps include samples with complete metabolomic profiles; Metabolites not detected in a given sample were excluded from analysis; therefore, n varies by metabolite bar plots include all samples in which the indicated metabolite was measured. *$p < 0.05$, **$p < 0.01$, ***$p < 0.001$, ****$p < 0.0001$; ns, $p > 0.05$.

Figure S6. Hepatic and splenic metabolic remodeling during GEMM KPC cachexia progression, related to Figure 3.

(A) Liver metabolite heatmap from control (CTRL; n = 5), pre-cachectic (PreCCX; n = 4), and severe cachexia (SCCX; n = 6) GEMM KPC mice, with selected metabolites shown individually.

(B) Principal-component analysis (PCA) of liver metabolomic profiles.

(C) Spleen metabolite heatmap from CTRL (n = 7), PreCCX (n = 5), and SCCX (n = 5) mice, with selected metabolites shown individually.

(D) PCA of spleen metabolomic profiles.

PreCCX, pre-cachectic; SCCX, severe cachexia; CCX, cachexia. Data are mean ± SEM where applicable. Selected metabolites were analyzed by ordinary one-way ANOVA with Dunnett's multiple-comparisons test versus CTRL. Undetected metabolites were excluded; therefore, n varies by metabolite. Heatmaps include samples with complete metabolomic profiles; Metabolites not detected in a given sample were excluded from analysis; therefore, n varies by metabolite bar plots include all samples in which the indicated metabolite was measured. *$p < 0.05$, **$p < 0.01$, ***$p < 0.001$, ****$p < 0.0001$; ns, $p > 0.05$.

bioRxiv preprint doi: https://doi.org/10.64898/2026.10.08.757616; this version posted October 9, 2026. The copyright holder for this preprint (which was not certified by peer review) is the author/funder, who has granted bioRxiv a license to display the preprint in perpetuity. It is made available under a CC-BY 4.0 International license.

Figure S7. Tryptophan pathway and metabolic responses across cachexia models, related to Figures 4–6.

(A–C) IDO1 and IL-6 expression in pancreas/tumor and liver as indicated.

(D and E) Serum and liver tryptophan-pathway metabolites in KP (D) and KL (E) lung cancer models relative to controls.

(F) Adenylate energy charge in soleus, liver, and spleen across CTRL, PreCCX, and SCCX stages.

(G–J) Metabolic-cage analysis of mixed-sex GEMM KPC mice treated with placebo ($n = 3$) or sustained-release fluoxetine ($n = 3$): cumulative food intake (G), wheel running (H), energy expenditure (I), and energy balance (J).

(K–M) Longitudinal body weight, fat mass, and lean mass (K), survival (L), and endpoint tumor weight (M) after fluoxetine treatment.

(N–Q) Metabolic-cage analysis of mixed-sex orthotopic PDAC-bearing mice treated with vehicle ($n = 5$) or 5-HTP ($n = 5$): cumulative food intake (N), wheel running (O), energy expenditure (P), and energy balance (Q).

(R–T) Longitudinal body weight, fat mass, and lean mass (R), survival (S), and endpoint tumor weight (T) after 5-HTP treatment.

(U) Circulating serotonin and serum metabolomic PCA in control ($n = 6$) and fluoxetine-treated ($n = 8$) orthotopic PDAC-bearing mice.

(V) Circulating kynurenine and serum metabolomic PCA in control ($n = 4$) and IDO1 inhibitor-treated ($n = 5$) orthotopic PDAC-bearing mice.

PreCCX, pre-cachectic; SCCX, severe cachexia. Gray bars indicate dark phases; shading denotes SEM. Data are mean ± SEM. Metabolic-cage data were analyzed by two-way repeated-measures ANOVA or mixed-effects models with Geisser–Greenhouse correction followed by Šídák's multiple-comparisons test. Statistics for other panels are indicated in the panels and STAR Methods. *$p < 0.05$, **$p < 0.01$, ***$p < 0.001$, ****$p < 0.0001$; ns, $p > 0.05$.
